# Effect of time-dependent dialysate bicarbonate concentrations on acid–base and uremic solute kinetics during hemodialysis treatments

**DOI:** 10.1038/s41598-024-52757-2

**Published:** 2024-01-28

**Authors:** Monika Wieliczko, Malgorzata Twardowska-Kawalec, Malgorzata Debowska, Mauro Pietribiasi, Urszula Bialonczyk, Jacek Waniewski, John K. Leypoldt, Joanna Matuszkiewicz-Rowinska, Jolanta Malyszko

**Affiliations:** 1 The Department of Nephrology, Dialysis and Internal Disease, Medical University of Warsaw, Warsaw, Poland; 2 Nalecz Institute of Biocybernetics and Biomedical Engineering, Polish Academy of Sciences, Warsaw, Poland

**Keywords:** Diseases, Nephrology

## Abstract

Recent studies have suggested benefits for time-dependent dialysate bicarbonate concentrations (D_bic_) during hemodialysis (HD). In this clinical trial, we compared for the first time in the same HD patients the effects of time-dependent changes with constant D_bic_ on acid–base and uremic solute kinetics. Blood acid–base and uremic solute concentration were measured in twenty chronic HD patients during 4-h treatments with A) constant D_bic_ of 35 mmol/L; B) D_bic_ of 35 mmol/L then 30 mmol/L; and C) D_bic_ of 30 mmol/L then 35 mmol/L (change of D_bic_ after two hours during Treatments B and C). Arterial blood samples were obtained predialysis, every hour during HD and one hour after HD, during second and third treatments of the week with each D_bic_ concentration profile. Blood bicarbonate concentration (blood [HCO_3_]) during Treatment C was lower only during the first three HD hours than in Treatment A. Overall blood [HCO_3_] was reduced during Treatment B in comparison to Treatment A at each time points. We conclude that a single change D_bic_ in the middle of HD can alter the rate of change in blood [HCO_3_] and pH during HD; time-dependent D_bic_ had no influence on uremic solute kinetics.

## Introduction

Continuous acid generation in thrice-weekly hemodialysis (HD) patients requires the addition of alkali or bases, typically bicarbonate anions (HCO_3_), to dialysis solutions at concentrations that exceed those in plasma or serum, resulting in a net alkali diffusive transfer to the patient. Based on this practice, KDOQI recommended predialytic serum HCO_3_ concentration (serum [HCO_3_]) greater than or equal to 22 mmol/L in chronic HD patients^1^, however, a more recent opinion by the same organization recommended to maintain serum HCO_3_ levels of 24–26 mmol/L^2^. Interestingly however, high predialytic blood bicarbonate concentration (blood [HCO_3_]) in dialysis patients likely caused by excessive delivery of HCO_3_ during HD resulting in postdialytic metabolic alkalosis associates with higher mortality^3^. It remains unclear how to optimize dialysate bicarbonate concentrations (D_bic_) to both neutralize interdialytic acid generation yet minimize postdialytic alkalosis^4–6^. 

Theoretical work has proposed that different dialysis solution base compositions^7^ or time-dependent D_bic_ profiles^8–11^ may provide improved delivery of alkali to the HD patient. And recently, a stepwise, linearly increasing D_bic_ profile was empirically evaluated in 20 chronic HD patients^12^. This latter study demonstrated that an increasing D_bic_ profile led to an approximately linear increase in intradialytic serum [HCO_3_] and achieved the same blood [HCO_3_] at the end of the HD treatment as was achieved in a previous study with a constant D_bic_^13^. This linear increase in blood [HCO_3_] resulted in a lower endogenous lactic acid production, a measure of organic acid production that has been proposed to be maladaptive and undesirable^5,7,13^. HD treatments with time-dependent D_bic_ profile can, however, only achieve the same predialytic blood [HCO_3_] as treatments with a constant D_bic_ if it delivers the same amount of HCO_3_ to the patient.

No previous clinical study has rigorously compared acid–base and uremic solute kinetics in the same HD patients using constant and time-dependent D_bic_. In this crossover clinical trial, we compared the effects of an increasing and decreasing D_bic_ during HD treatments on acid–base kinetics. As changes in blood and extracellular acid–base status may alter the kinetics of important uremic solutes, we also assessed the effects of increasing and decreasing D_bic_ on the kinetics of urea and creatinine. Acid–base and uremic solute kinetics were assessed by concentration changes in blood during the treatment and one hour after the treatment.

## Material and methods

### Patients

Twenty patients from the Dialysis Unit at the Medical University of Warsaw completed the study protocol. The study inclusion criteria were patients who were over 18 years of age, in stable condition, and had been treated by chronic HD for at least three months with an arterio-venous fistula. The study exclusion criteria were patients who were diabetic, had inflammation, had other debilitating diseases, or were taking Sevelamer during the previous month. None of the patients were taking oral bicarbonates. A total of 120 HD treatments were monitored.

### HD treatments

All patients were treated 3 times per week, and all HD treatments were four hours in duration. The Fresenius 4008S dialysis delivery systems were used for setting constant blood and dialysate flow rates and the delivery of dialysis solution. Low flux hemodialyzers were used in all but 7 patients. All dialysis solutions contained acetate at a concentration of 3 mEq/L. The hemodialyzer and blood flow rate were individualized for each patient and were generally those used during routine HD treatments; the dialysate flow rate was always 500 mL/min. Fluid removal or ultrafiltration rates were individually prescribed to achieve a target postdialytic weight.

### Study interventions

Patients were studied during 4 consecutive weeks in two groups (1 and 2) using a non-randomized protocol: the first 11 patients were in Group 1 and the latter 9 patients were in Group 2. One week before the study, all patients were treated with a constant D_bic_ of 35 mmol/L to have a stable acid–base status at the beginning of the study. For all patients, Week 1 consisted of 3 HD treatments with a constant D_bic_ of 35 mmol/L; this control treatment was termed Treatment A. Group 1 patients were then treated by 3 HD treatments during Week 2 with a D_bic_ of 35 mmol/L for the first two hours followed by 30 mmol/L for the remainder of the treatment; this intervention was called Treatment B. For all patients, Week 3 consisted of 3 HD treatments with a D_bic_ of 35 mmol/L (wash-out period). During Week 4 Group 1 patients were then treated by 3 HD treatments with a D_bic_ of 30 mmol/L for the first two hours followed by 35 mmol/L for the remainder of the treatment termed Treatment C. Group 2 patients were instead treated by Treatment C during Week 2 and Treatment B during Week 4.

### Study measurements

Measurements were made during the second and third HD treatments during Weeks 1, 2 and 4. Blood samples were obtained from the arterial-venous fistula before the start of the treatment and (for 19 patients) one hour after the end of the treatment (post-dialytic rebound sample) and from blood flowing into the arterial port of the hemodialyzer every hour during the treatment. Each sample was used to determine blood partial pressure of carbon dioxide (pCO_2_) content and pH using a blood gas analyzer (Radiometer ABL 90 Version 3.4 M2); the blood [HCO_3_] of each sample was then calculated using the Henderson-Hasselbalch equation. Each blood sample was also used to determine the plasma concentration of urea and creatinine (not at 1 and 3 h of treatment) using the clinical analyzers Cobas Roche within the Medical University of Warsaw.

The blood pressure, heart rate and saturation were monitored each hour during the entire dialysis treatment and one hour after it.

All experimental protocols were approved by the Bioethics Committee at the Medical University of Warsaw (No KB/91/2018) and written, informed consent, approved by this Bioethics Committee, was obtained for each patient prior to participation in the study. All methods were in accordance with ethical standards of the responsible committee on human experimentation and with the Helsinki Declaration of 1975, as revised in 2013. The study has been registered in ClinicalTrials.gov NCT05861700.

### Statistical analysis

Time-dependent changes in blood concentration measurements were first averaged for the second and third HD treatments during each week. Linear mixed models were used to formally assess the changes in concentration levels. The random effect included in the model were subject-specific while the fixed effects variables were Treatment type (A, B or C), time of measurement during treatment and the interaction between Treatment type and time. The latter part of the model allows to test for statistical significance whether the existing differences between treatments remain constant throughout the time of measurement or whether the effect of treatment changes over time. Statistical analyses were also performed with Treatment Group (1 or 2) as an additional fixed effects variable, but those analyses failed to demonstrate a statistically significant difference between Group 1 and 2 results. (Note that this was not strictly true when analyzing time-dependent changes in blood [HCO_3_] where a significant effect of group, P = 0.026, was observed; however, the addition of treatment Group to the statistical model did not alter any effect size of the interaction terms or any other statistical parameter.) Thus, time-dependent changes in measured variables were reported below for each Treatment type for Groups 1 and 2 combined. All statistical analyses for blood acidity were performed on the hydrogen ion concentration since pH is a logarithm transformed concentration^14^; however, the results are displayed in pH units.

The difference between the end of the treatment and 1-h postdialytic blood [HCO_3_] and hydrogen ion concentrations was calculated as a percentage change in the concentration levels between these two consecutive time labels. The significance of the percentage change in postdialytic value as well as the Treatment type was assessed using linear mixed models with Treatment type as a fixed effect, subject-specific random effect, and the percentage change in concentration level as a dependent variable.

A P-value of less than 0.05 was considered statistically significant.

### HCO_3_ delivery to patient

HCO_3_ delivery to the patient was calculated as the time integration of the HCO_3_ flux equation, including both diffusive and convective transport as described by Sargent et al.^13^. Since no measurements were made of acetate concentration in blood or the dialysis fluids, we acknowledge that delivery of HCO_3_ significantly underestimates total delivery of alkali to the patients^13^.

### Uremic solute kinetics and small solutes

Statistical analysis of time-dependent changes in urea and creatinine concentrations were performed as above. Single-pool urea Kt/V (spKt/V) was calculated from the predialytic and immediate postdialytic (4-h) urea concentrations using the 2nd-generation Daugirdas formula^15,16^. Equilibrated urea Kt/V (eKt/V) was calculated from the predialytic and the 1-h postdialytic (5-h) urea concentrations using the same formula^17,18^.

All time-dependent blood concentration results are presented in tables and figures as mean values only for clarity.

## Results

Of the 20 HD patients who participated in this clinical trial, 12 were male; patient age was 65 ± 11 (SD) years. Their height was 169.9 ± 9.8 cm while their body mass index was 25.6 ± 3.1. Predialytic body weight and ultrafiltration volume during the second HD treatment of Week 1 were 75.3 ± 11.3 kg and 1.9 ± 1.0 L, respectively. There were no differences between Group 1 and 2 concerning anthropometric variables and dialysis settings (aside from Treatment protocol). There were no statistically significant differences in blood [HCO_3_], blood pH, pCO_2_, urea and creatinine concentrations between treatments with low and high flux hemodialyzers.

### HCO_3_ concentration

**Figure 1:**
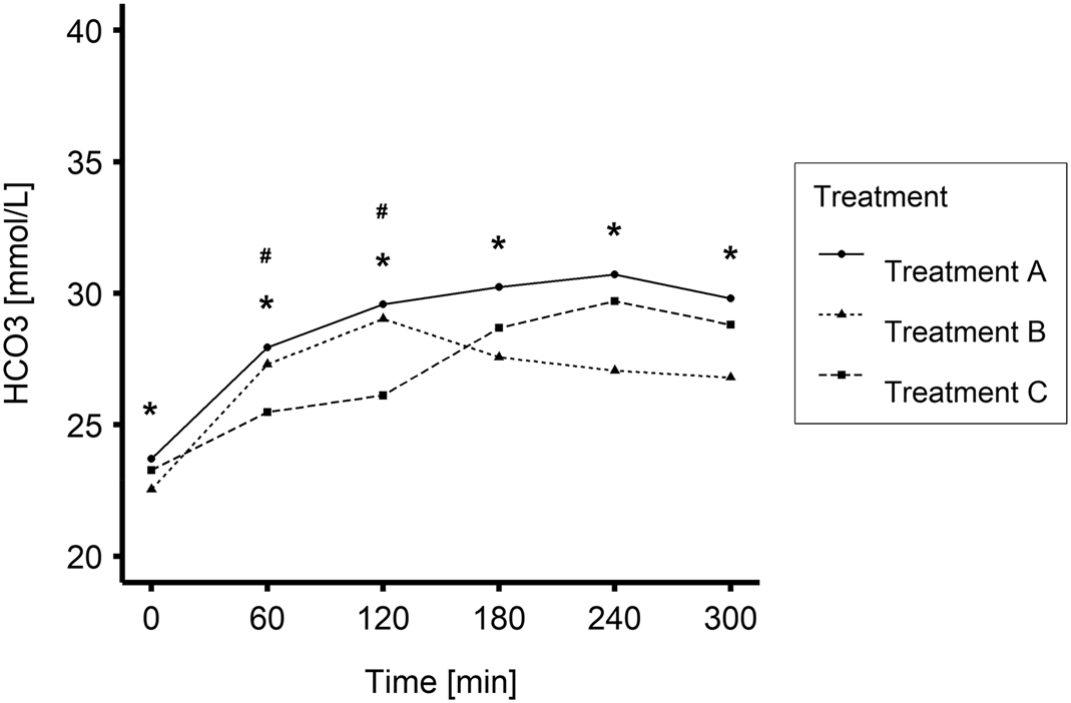
Mean values of blood bicarbonate concentration ([HCO_3_]) during Treatments A, B and C (0–240 min N = 20; 300 min N = 19). *P < 0.05 Treatment B vs Treatment A; ^#^P < 0.05 Treatment C vs Treatment A.

Time-dependent changes in blood [HCO_3_] for Treatments A, B and C are plotted in Fig. 1 and presented in Table 1.

**Table 1:** Mean ± standard deviations for blood [HCO_3_], pCO_2_ and pH as a function of time during different treatment interventions for Group 1 and 2 patients (0–240 min N = 20; 300 min N = 19; *P < 0.05 vs Treatment A).

Time (mins)	Treatment A	Treatment B	Treatment C
[HCO_3_] (mmol/L)			
0	23.7 ± 1.7	22.4 ± 2.5*	23.3 ± 2.1
60	27.9 ± 1.6	27.3 ± 1.7*	25.5 ± 1.7*
120	29.6 ± 1.5	29.0 ± 2.0*	26.1 ± 1.5*
180	30.2 ± 1.5	27.5 ± 1.6*	28.7 ± 1.8
240	30.7 ± 1.5	27.1 ± 2.0*	29.7 ± 1.7
300	29.8 ± 1.7	26.8 ± 1.5*	28.8 ± 1.5
[pH]			
0	7.41 ± 0.03	7.40 ± 0.04*	7.40 ± 0.03
60	7.46 ± 0.03	7.45 ± 0.04*	7.43 ± 0.02*
120	7.48 ± 0.03	7.48 ± 0.03	7.44 ± 0.03*
180	7.50 ± 0.03	7.47 ± 0.04*	7.48 ± 0.03
240	7.51 ± 0.03	7.48 ± 0.03*	7.503 ± 0.02
300	7.50 ± 0.03	7.47 ± 0.03*	7.49 ± 0.03
[pCO_2_] (mmHg)			
0	37.4 ± 3.1	37.2 ± 2.9*	37.5 ± 3.2
60	39.3 ± 2.9	38.7 ± 3.2*	38.8 ± 3.0
120	39.9 ± 3.1	39.0 ± 4.5*	38.7 ± 3.1
180	39.0 ± 2.5	38.1 ± 3.3*	39.8 ± 3.3
240	38.6 ± 2.6	37.1 ± 2.3*	38.4 ± 2.6
300	38.0 ± 2.8	37.3 ± 2.5*	38.0 ± 2.7

Statistical analysis showed significant differences in the mean blood [HCO_3_] between Treatments A and B for each measurement taken during the session (0, 60, 120, 180, 240, 300 min) (P < 0.005). Treatment C differed significantly from Treatment A in terms of mean blood [HCO_3_] during the first 3 h of HD. There was also a significant effect of time that was independent of the chosen Treatment (P < 0.001).

When the D_bic_ was held constant at 35 mmol/L (Treatment A), blood [HCO_3_] increased during the first two to three hours and then remained relatively constant. When the D_bic_ was 35 mmol/L during the first two hours of HD treatment and then reduced to 30 mmol/L for the remainder of the treatment (Treatment B), the blood [HCO_3_] increased a lot (similar to Treatment A) during the first two hours of treatment, as expected and then decreased to levels below those in Treatment A. In contrast, when the D_bic_ was 30 mmol/L during the first two hours of HD treatment, after which it was increased to 35 mmol/L (Treatment C), the blood [HCO_3_] did not increase as rapidly during the first 2 h as in Treatments A and B, but then increased significantly during the last 2 h of treatment. Thus, as expected during Treatment C, there was a more gradual rise in the intradialytic blood [HCO_3_] than during Treatment A during the first three hours, and the blood [HCO_3_] at the end of Treatments C and A were not significantly different. The intradialytic changes in blood [HCO_3_] shown in Fig. 1 have been previously evaluated quantitatively using the hydrogen ion mobilization model in a separate publication^19^: the results from that study demonstrated that such changes in blood [HCO_3_] are those predicted by the model with a mobilization parameter independent of the D_bic_ profile.

### pH

**Figure 2:**
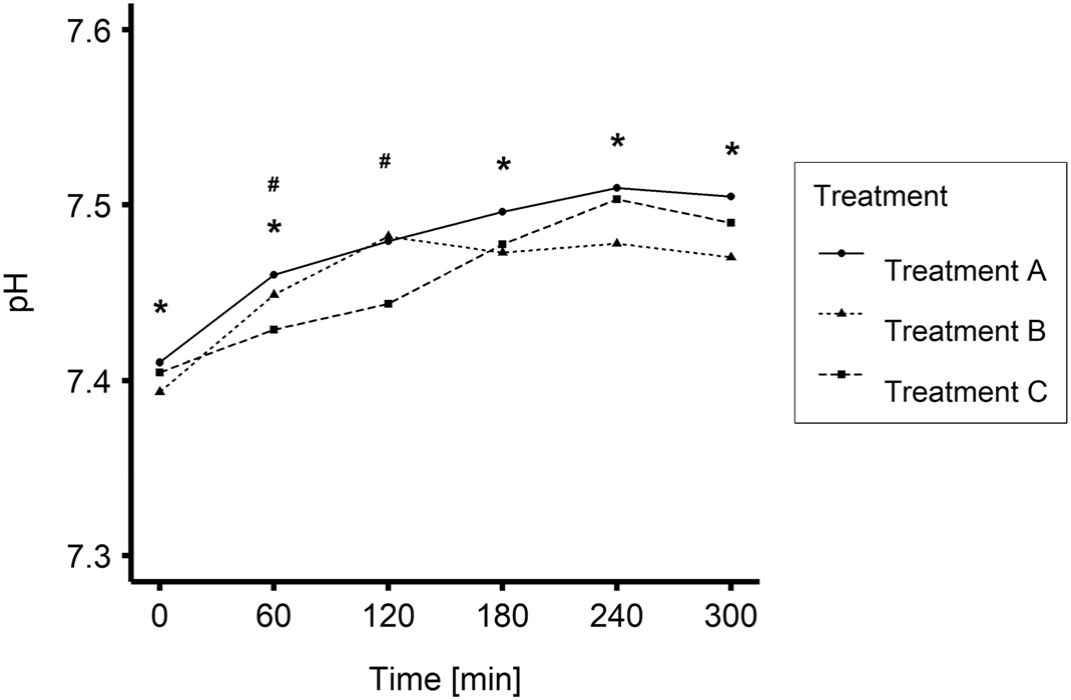
Mean values of blood pH during Treatments A, B, and (0–240 min N = 20; 300 min N = 19). *P < 0.05 Treatment B vs Treatment A; ^#^P < 0.05 Treatment C vs Treatment A.

Time-dependent changes in blood pH for Treatments A, B and C are plotted in Fig. 2 and presented in Table 1. There was a significant difference between Treatment B and Treatment A for values measured during 0, 60, 180, 240 and 300 min of HD (P < 0.005). Mean levels of pH differed between Treatment C and Treatment A during the first two hours of HD (P < 0.01).

### pCO_2_

**Figure 3:**
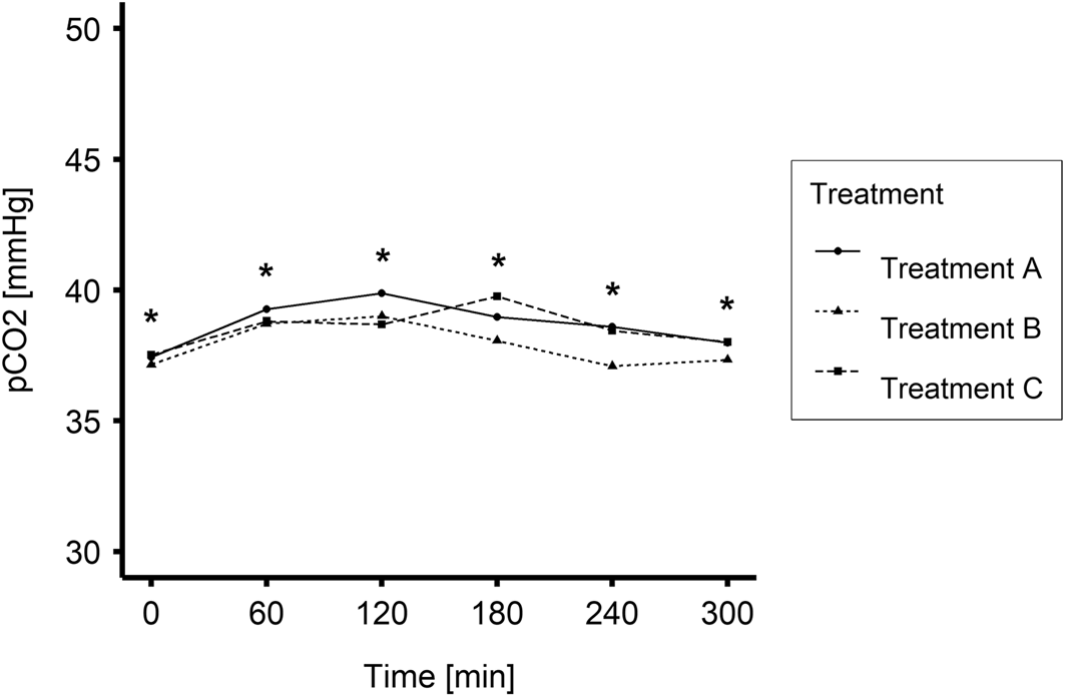
Mean values of blood partial pressure of carbon dioxide (pCO_2_) during Treatments A, B, and C (0–240 min N = 20; 300 min N = 19). *P < 0.05 Treatment B vs Treatment A.

Time-dependent changes in blood pCO_2_ for Treatments A, B and C are plotted in Fig. 3 and presented in Table 1. There were significant differences between Treatments A and B (P < 0.001) but the interaction term between treatment and time was insignificant. Thus, there was no evidence that the effect of Treatment B changes over time; there was a significant effect of time during the treatment (P < 0.05) but not postdialysis. Hence, there was no evidence that the levels of pCO_2_ differed pre and post dialysis.

### Postdialytic changes in acid–base concentration

Postdialytic changes in blood [HCO_3_] were observed between the values measured at the end of HD and one hour after in all treatments (P < 0.001). We found significant differences in the percentage change in blood [HCO_3_] at the end of dialysis and one hour after between Treatments A and B (P = 0.01) with Treatment B lower than Treatment A. There was no significant difference in the percentage changes in postdialytic values between Treatment A and C (P = 0.76).

Postdialytic changes were not observed for blood pH (P = 0.07) and pCO_2_ (P = 0.12) levels. The percentage changes in these variables after the first hour after dialysis are reported in Table 2.

**Table 2:** Mean ± standard deviations of percentage post-dialytic changes in serum [HCO_3_], pH, pCO_2_ (1 h after the end of HD) for each treatment (N = 19).

	Treatment A	Treatment B	Treatment C
[HCO_3_]	3.02 ± 2.97%	0.84 ± 3.92% (P = 0.01)*	2.84 ± 3.18% (P = 0.76)
pH	0.07 ± 0.24%	0.11 ± 0.26% (P = 0.07)	0.16 ± 0.24% (P = 0.07)
pCO_2_	1.65 ± 6.17%	− 1.06 ± 6.6% (P = 0.12)	− 0.01 ± 6.31% (P = 0.12)

*P < 0.05 vs. Treatment A.

### Acid–base parameters before HD

The lowest blood [HCO_3_] before HD was found in Treatment B and this concentration was statistically significant lower in Treatment B than in Treatment A (mean 22.4 ± 2.5 mmol/L vs 23.7 ± 2.01 mmol/L, respectively, P = 0.006). Predialysis blood [HCO_3_] values for Treatment C did not statistically differ from those before Treatment A.

We found 13 patients, who had blood [HCO_3_] before HD less than 22 mmol/L in all Treatments: it was 3 patients (15%) in Treatment A, 6 patients (30%) in Treatment B and 4 patients (20%) in Treatment C. The Cochran Q test did not find significant differences in the numbers of patients with blood [HCO_3_] < 22 mmol/L pre-HD. However, in our study, the fraction of patients with blood [HCO_3_] < 22 mmol/L pre-HD was the highest in Treatment B.

Both predialytic pH and pCO_2_ levels differed significantly between Treatment A and B and they levels were significantly lower before HD for Treatment B than Treatment A (both P < 0.01).

### HCO_3_ delivery

Delivery of HCO_3_ to the patient during Treatments A, B and C were 159 ± 68 (SD) mmol/L, 151 ± 84 mmol/L, and 134 ± 80 mmol/L respectively; these values did not differ statistically (P = 0.27), although the mean delivery in Treatment C was the lowest. As noted above, these calculated values significantly underestimate total alkali delivery to the patient because they omit the contribution from acetate transfer to the patient.

### Uremic solute kinetics and small solutes

**Figure 4:**
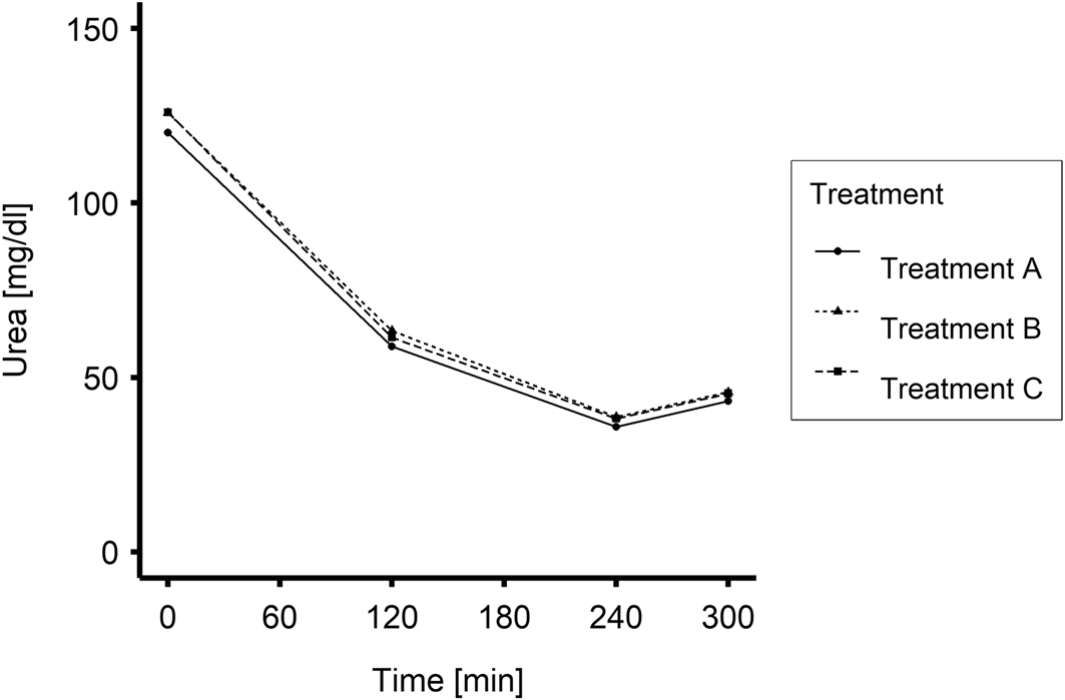
Mean values of urea concentration during Treatments A, B and C (0–240 min N = 20; 300 min N = 19).

**Figure 5:**
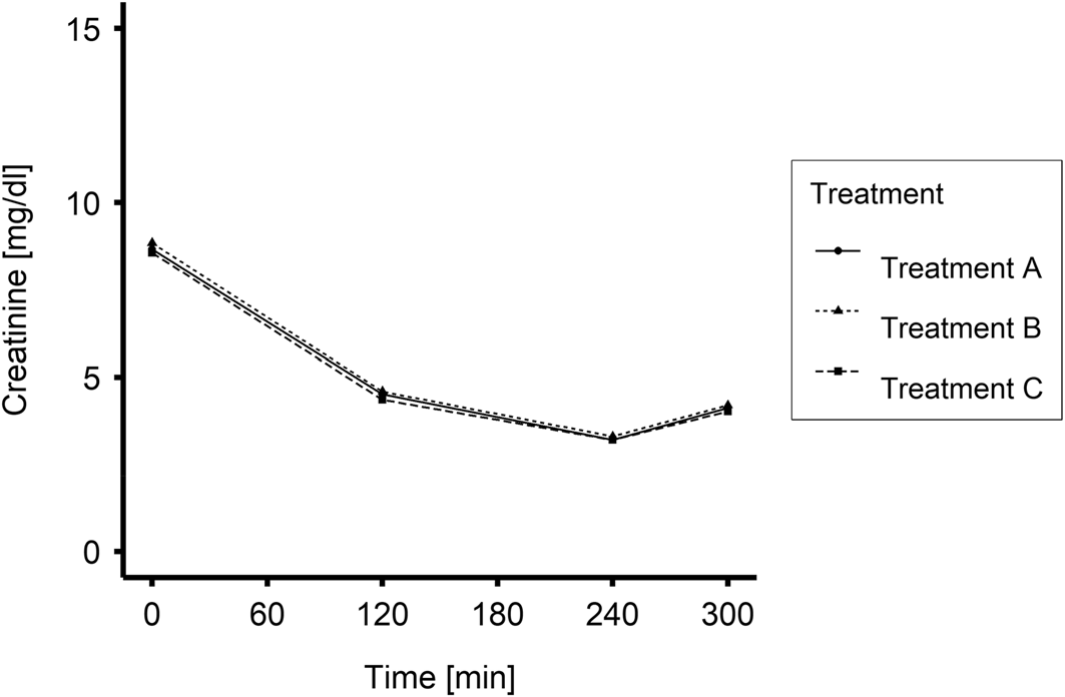
Mean values of blood creatinine concentration during Treatments A, B and C (0–240 min N = 20; 300 min N = 19).

Time-dependent changes in plasma urea and creatinine concentrations for Treatments A, B and C are plotted in Figs. 4 and 5, respectively. There were no statistically significant differences between Treatment B or C and Treatment A. As expected, the plasma concentration of both solutes decreased significantly during the treatment; these findings were not statistically different for Treatments A, B and C. Further, postdialytic solute concentrations changed to higher levels for the uremic solutes (nonzero postdialytic increase P < 0.001) during all treatments. However, although with mean values numerically similar, creatinine rebound in Treatment C was significantly lower than in Treatment A (P = 0.02).

Values of spKt/V and eKt/V for Treatment A were 1.43 ± 0.22 and 1.23 ± 0.21, respectively; for Treatment B were 1.40 ± 0.19 and 1.21 ± 0.16, respectively; and for Treatment C were 1.40 ± 0.17 and 1.21 ± 0.16, respectively. There were no statistically significant differences in spKt/V and eKt/V between treatments.

The measured concentrations of sodium, chloride and the total osmolarity before and after HD are reported in Table 3.

**Table 3:** Mean ± standard deviations of sodium and chloride concentrations, and total osmolarity measured with the blood gas analyzer.

Time (mins)	Treatment A	Treatment B	Treatment C
Sodium (mmol/L)			
0	139.3 ± 2.7	140.4 ± 2.5*	139.1 ± 3.2
240	138.9 ± 1.7	138.9 ± 1.5	138.3 ± 2.1
300	138.7 ± 1.8	138.8 ± 1.5	138.2 ± 2.1
Chloride (mmol/L)			
0	101.6 ± 3.1	104.0 ± 3.4*	102.4 ± 3.7
240	97.8 ± 2.1	101.7 ± 1.7*	98.9 ± 2.2*
300	98.1 ± 2.4	102.3 ± 2.8*	99.8 ± 2.3*
Total osmolarity (mmol/L)			
0	285.2 ± 5.5	287.1 ± 4.8	284.7 ± 6.4
240	282.9 ± 3.6	283.1 ± 3.4	281.8 ± 4.3*
300	282.7 ± 3.8	282.6 ± 3.4	281.7 ± 4.6
Serum albumin (g/dL)			
0	4.15 ± 0.30	4.02 ± 0.34*	4.06 ± 0.33*
Electrolytes concentration in dialysate at T = 0 (mmol/L)			
Sodium	138.40 ± 1.13		
Potassium	2.35 ± 0.58		
Calcium	1.34 ± 0.12		

Measurements in Group 1 and 2 and sessions HD2 and HD3 were pooled together (N = 39: 0–240 min N = 20; 300 min N = 19; *P < 0.05 vs Treatment A).

### Blood pressure, heart rate, saturation

We did not note any statistically significant changes in blood pressure, heart rate and saturation, the patient’s well-being remained good throughout all treatments.

## Discussion

This crossover clinical trial is the first to directly compare acid–base and uremic solute kinetics during time-dependent and constant D_bic_ in the same HD patients. When D_bic_ was held constant at 35 mmol/L (Treatment A), blood [HCO_3_] increased during the first two to three hours and then remained relatively constant. This kinetic behavior is consistent with data from previous clinical studies using a constant D_bic_^13,20^. Lowering D_bic_ concentration during the treatment from 35 to 30 mmol/L resulted in lower blood [HCO_3_] at the end of and 1 h after the treatment, but also before HD. In contrast, raising D_bic_ from an initially lower dialysate [HCO_3_] (30 to 35 mmol/L) resulted in a blood [HCO_3_] like that if D_bic_ was held constant at the higher level, also before HD, to which the KDOQI recommendation apply^1^. This latter finding was suggested in a previous clinical study where D_bic_ was increased in 8 steps during the HD treatment^12^. The current study suggests that a simple protocol with D_bic_ increasing only once during the HD treatment may be a practical approach to prescribing a time-dependent D_bic_ as suggested in a recent review^5^.

The above changes in intradialytic blood [HCO_3_] were paralleled by qualitatively similar intradialytic changes in blood pH, although some of the differences from alternative D_bic_ profile were not statistically significant. Such findings are to be expected if equilibrium conditions apply and blood pCO_2_ is relatively constant. We note that some differences in blood pCO_2_ were observed in the current study; however, those differences were small and not clinically significant. Maintenance of a relatively constant blood pCO_2_ during HD treatments despite a large influx to the patients of HCO_3_ and subsequent conversion to carbon dioxide is known to be mitigated by the increase in minute ventilation^21^.

Postdialytic reduction in blood [HCO_3_] and pH in the current study are consistent with the recent studies by Park et al.^20^. The latter study is the only publication where postdialytic changes in acid–base levels were considered, but those investigations were limited to the use of a constant D_bic_. The current study is the first to demonstrate that postdialytic changes in blood [HCO_3_] and pH can also occur when using a time-dependent D_bic_ profile; thus the combination of the current and the previous study demonstrate that postdialytic changes in acid–base parameters should be considered in future studies.

There are potential clinical advantages and disadvantages to time-dependent D_bic_ profile during routine HD treatments; however, they remain to be tested to date in clinical studies. As mentioned above, an increasing D_bic_ profile results in a more gradual increase in blood [HCO_3_] that may lower the production of endogenous organic acid, proposed to be maladaptive and undesirable^5,7,13^. If a time-dependent D_bic_ leads to reduced alkali delivery of HCO_3_ to the patients, however, this would likely lead to a reduction in predialytic blood [HCO_3_] and metabolic acidosis in subsequent HD treatments as the patient reaches a new steady state acid–base balance. Treatment C in our study with increasing D_bic_ from 30 to 35 mmol/L in the middle of 4-h HD session showed, that the blood [HCO_3_] before and at the end of HD is no different from a protocol based on the continuous supply of larger amounts of bicarbonate (with constant D_bic_ 35 mmol/l—Treatment A) and meets the KDOQI recommendation in a similar percentage, and HCO_3_ delivery is slightly less.

The lack of an effect of a time-dependent D_bic_ profile on urea and creatinine kinetics was not unexpected as these uremic solutes do not carry an electric charge, but the current study provides clinical data to support the contention that the removal of neutral uremic solutes will not be altered when using time-dependent D_bic_ profile. Further studies are necessary to assess whether uremic solutes that carry an electric charge will also not be influenced by the use time-dependent D_bic_ profiles. Finally, our results suggest that urea Kt/V parameters remain an accurate assessment of the dose of HD when using time-dependent D_bic_ profiles and uremic solute removal is not adversely affected by the modified D_bic_ protocols. Although some pre- and post- dialysis values of sodium and chloride concentration were different in Treatments B and C compared to Treatment A (Table 3 P < 0.05), their little magnitude including the mostly non-significant changes in total osmolarity, suggest that time-dependent D_bic_ profile did not alter water and ionic solute transport in a clinically significant way, compared to a standard treatment.

This study is not without limitations. First, the crossover design was not randomized. Second, the study site was a single university dialysis unit; thus, the results may not be generalized to other dialysis facilities. Third, the calculation of HCO_3_ delivery to the patient resulted in significant variability because HCO_3_ transfer was largely by diffusion and thus was dependent on small differences between the blood and dialysate [HCO_3_]. Fourth, no measurements of blood and dialysate acetate concentration were made so total alkali delivery to the patient could not be calculated. Note however that all dialysate fluids contained the same concentration of acetate so that the contribution of acetate to acid–base balance should be similar among the study treatments in this study.

## Conclusion

We conclude that a single change of D_bic_ in the middle of HD can alter the rate of change in blood [HCO_3_] and pH during the HD without affecting the stability of the circulatory system. Time-dependent D_bic_ had no influence on uremic solute kinetics. A longitudinal study should be conducted to confirm this result.

## Data Availability

The data supporting the findings of this study are not currently available in a public repository but can be made available upon request to the corresponding author.
